# Protein appetite as an integrator in the obesity system: the protein leverage hypothesis

**DOI:** 10.1098/rstb.2022.0212

**Published:** 2023-10-23

**Authors:** David Raubenheimer, Stephen J. Simpson

**Affiliations:** Charles Perkins Centre and School of Life and Environmental Sciences, The University of Sydney, Sydney, New South Wales 2006, Australia

**Keywords:** nutrition, protein leverage, obesity, ultra-processed foods‌

## Abstract

Despite the large volume and extensive range of obesity research, there is substantial disagreement on the causes and effective preventative strategies. We suggest the field will benefit from greater emphasis on integrative approaches that examine how various potential contributors interact, rather than regarding them as competing explanations. We demonstrate the application of nutritional geometry, a multi-nutrient integrative framework developed in the ecological sciences, to obesity research. Such studies have shown that humans, like many other species, regulate protein intake more strongly than other dietary components, and consequently if dietary protein is diluted there is a compensatory increase in food intake—a process called protein leverage. The protein leverage hypothesis (PLH) proposes that the dilution of protein in modern food supplies by fat and carbohydrate-rich highly processed foods has resulted in increased energy intake through protein leverage. We present evidence for the PLH from a variety of sources (mechanistic, experimental and observational), and show that this mechanism is compatible with many other findings and theories in obesity research.

This article is part of a discussion meeting issue ‘Causes of obesity: theories, conjectures and evidence (Part II)’.

## 1. Introduction

The global rise in obesity is both among the simplest and most complex of issues in public health. It is, on the one hand, straightforwardly true that excess body fat can accumulate only if more energy is eaten than is expended. On the other hand, an immense amount of research has produced a plethora of information and theories, but there is little consensus about why such energy imbalance develops or what to do about it [1].

The breadth spanned by the papers in this special issue, and other perspectives, attests to a vibrant, multi-disciplinary obesity research community. However, the lack of consensus and failure to tackle what has been described by the World Health Organization as the largest health threat facing mankind raises the question of what is limiting progress in the field. It is, of course, inevitable that some results cannot be replicated, some theories will turn out not to be useful, and that important discoveries await further research. We believe, however, that a more fundamental issue is a tendency to regard existing perspectives on obesity as separate, unrelated or even competing explanations [2], rather than potentially inter-related co-contributors to the problem in a complex ecosystem of interacting biological, behavioural, cultural and societal factors.

An overarching aim in this paper is to advocate for the more widespread and explicit adoption of integrative perspectives in obesity research. Integrative perspectives reflect the reality of a problem that has emerged from a complex system comprising components from the molecular to behaviour, culture, global trade and geopolitics, and emphasize potential connections and interactions among the many relevant research approaches, rather than their differences and incompatibilities. Importantly, integrative framing can help deal with the fundamental but vexatious issue in obesity research of defining the key concept of ‘causation’ [3,4], through recognizing that systems outcomes have many interacting causes and what is considered ‘the cause’ or ‘primary driver’ of obesity depends both on the question being asked and the context for asking it. For example, it is equally true that obesity is ‘caused’ by energy imbalance and the aggressive marketing of ultra-processed foods (UPF) [5] and it would be futile to argue that one or the other is ‘responsible’. However, it would not be futile to consider which cause is most relevant to the rise of the obesity epidemic, or to inter-individual variation in susceptibility to obesity.

We present an integrative framework derived from the ecological sciences, nutritional ecology, and a specific integrative tool, nutritional geometry, and demonstrate how these have been applied to examine the causes of obesity. A core contribution of nutritional ecology to obesity research is the phenomenon of protein leverage, in which the strong human appetite for protein drives increased intake when dietary protein is diluted [6,7]. We will first introduce our ecological systems framework and then review evidence for protein leverage and its relevance for obesity. The fact that this evidence is consistent across cellular signalling mechanisms, randomized control trials and population studies suggests the capacity of protein leverage to integrate across organizational levels and scales within the obesity system. In the final section, we explicitly demonstrate this integrative capacity through examining potential links between protein leverage and diverse issues in obesity research, including several of the issues and perspectives discussed in other contributions to this volume.

## 2. Nutritional ecology and nutritional geometry

The field of nutritional ecology is an integration of nutritional and ecological sciences that developed explicitly to focus on the interface between biological and ecological aspects of nutrition [8–10]. To deal with the complexity of this interface, nutritional ecology has been framed in systems thinking where the core entities are the organism, the environment, diet and their respective components and interactions [11]. Developed in the context of non-human animals, the nutritional ecology framework is increasingly being applied to humans [12,13].

Nutritional geometry is an analytical framework (henceforth NGF) for examining how biology interfaces with food environments via nutrition [10,14]. Since the general logic of NGF has been described in several publications (most recently [15]), here we restrict our discussion to some key points that predispose this approach to integrative analysis. At the core of NGF models is a geometric representation of nutrients and other dietary components, which is multi-dimensional (i.e. mixture-based) and can thus partition the individual and interactive (e.g. synergistic or modulating) effects of these components. In addition to this horizontal integration of dietary components, mixtures are modelled hierarchically in that components can be decomposed into sub-components (e.g. dietary carbohydrate into its sub-categories) [16] or integrated into higher-level mixtures (e.g. macronutrients into foods, foods into meals, meals into diet and ultimately dietary patterns) [12]. Adjunct variables that are nominally associated with dietary mixtures but are expressed in different units (e.g. expression levels of a gene, titres of a hormone, energy intakes or body composition) can be represented in geometric models as response surfaces, where the topography describes the parameters of the association. These properties—horizontal integration, vertical integration and incorporation of adjunct variables—make NGF well suited to building integrative models that encompass interactions among nutrients and biological and environmental factors relevant to nutrition.

## 3. The power of protein

**Figure 1.**
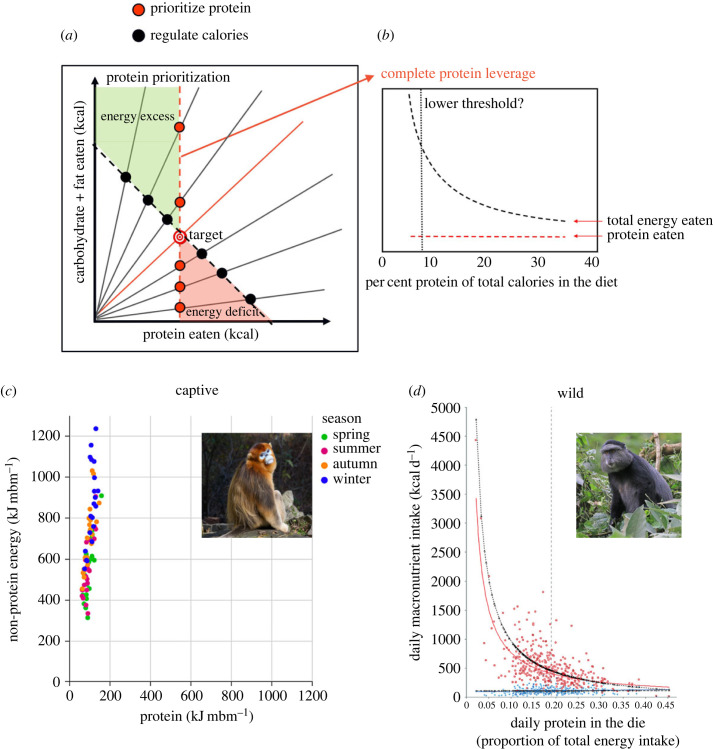
Theoretical depiction of the protein prioritization pattern of macronutrient regulation and empirical examples from non-human primates. (*a*) The red target (intake target) represents the macronutrient composition of the diet which the feeding regulatory systems compose in balanced food environments, and the red radial line shows the macronutrient ratio of a food that contains the nutrients in the target balance. Black radials show macronutrient imbalanced diets that are too low (top left) or too high (bottom right) in energy from protein relative to fat and carbohydrates. The vertical dashed red line shows ‘protein prioritization’, in which the regulatory systems maintain protein intake constant at the target level when eating macronutrient imbalanced diets, and consequently over-eat or undereat fat and carbohydrate (hence total energy) relative to the target intakes on low- and high-protein diets (protein leverage). The black diagonal dashed line shows the regulatory pattern in which there is no protein leverage—in this case, energy intake is maintained constant across the range of dietary macronutrient ratios. Protein leverage need not be complete (protein intake constant), but partial, in which there are smaller energy excesses (green area) and deficits (pink area) (modified from [7]). (*b*) A different depiction of complete protein leverage, shows that if protein intake is maintained constant (complete protein leverage) energy intake increases exponentially with decreasing dietary percentage protein (modified from [6]). (*c*) Captive golden snub-nosed monkeys (*Rhinopithecus roxellana*) show protein prioritization both across seasons (colours) and within seasons (from [17]). (*d*) In Kenyan blue monkeys (*Cercopithecus mitis*) macronutrient intakes conform closely to the predictions of complete protein prioritization. Blue symbols, observed daily protein intakes; red symbols, observed daily non-protein energy intake; black exponential curve, predicted non-protein energy intake under complete protein leverage; red exponential curve, regression for observed non-protein intakes. Vertical dashed line represents the mean proportion of protein to non-protein energy (modified from [18]). Credit goes to: Charles J. Sharp/Wikimedia Commons.

NGF studies have demonstrated that protein is a particularly influential component in the nutritional ecology of taxa from insects to primates [14]. Many species of non-human primates, for example, maintain daily protein intakes within narrow limits, allowing fat and carbohydrate to vary more widely with ecologically imposed variation in the macronutrient ratios of available diets—a pattern of macronutrient regulation termed ‘protein prioritization’ (figure 1*a*,*b*). Known instances include spider monkeys [19], black howler monkeys [20], golden snub-nosed monkeys [17] (figure 1*d*), Kenyan blue monkeys [18] (figure 1*d*), black-and-white ruffed lemurs [21], orangutans [22] and chimpanzees [23].

Parenthetically, as shown in figure 1*a,c* versus *b,d*, two graphical formats are used for examining protein prioritization, which are interconvertible but provide different insights. Bi-coordinate intake plots (figure 1*a*,*c*) are especially helpful in demonstrating asymmetrical intake patterns, for example where protein prioritization occurs on one side of the intake target but not on the other (discussed in [7]). We have documented numerous examples of asymmetrical prioritization patterns previously in comparative studies across species [14]. Fitting a power function across the range of dietary percentage protein (figure 1*b*,*d*) shows the nonlinear relationship between dietary protein density and absolute intakes and provides a graphical depiction of the power regression model used to test the strength of protein prioritization [7,24]. In figure 1*b*,*d*, absolute intakes of macronutrient energy are plotted on the *y*-axis, but it is also instructive to plot absolute intakes in relation to food bulk (g). As discussed further below, this enables the interactive effect of protein regulation and energy density on energy intake to be modelled.

In humans, the rising prevalence of obesity across the past 60 years has been attributed principally to excess energy intake rather than a decline in energy expenditure (although see [25]). Fats and carbohydrates have provided the major source of these excess calories, with the relative contributions of these two macronutrients varying across populations. Meanwhile, whereas the food sources of dietary protein have changed over time, protein intake has remained much more stable, both as a percentage of total energy intake and in terms of absolute amounts eaten [6,24,26–28]. In the most proximate sense, therefore, excess calories from protein have not caused global obesity. Paradoxically, however, an integrative systems perspective suggests that the very constancy of protein intake indicates that protein may have played a fundamental role in causing obesity via its interaction with other system components.

**Figure 2.**
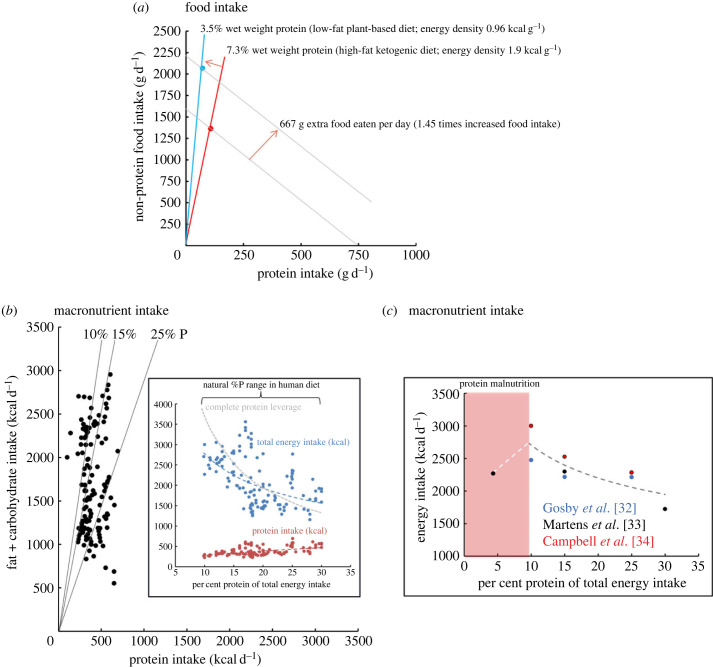
(*a*) An example showing that protein leverages food intake, with energy intake following as a function of dietary energy density. Data are replotted from [29], in which subjects were provided ad libitum access to a low-fat, plant-based diet and a high-fat, ketogenic diet for 14 days each. Diets differed in energy density (0.96 versus 1.9 kcal g^−1^ for plant-based and ketogenic diets, respectively) and, although near matched for protein as a per cent of total energy (14.2 versus 15.7%), they differed in protein density per gram of food (3.5 versus 7.3% protein g^−1^, for plant-based and ketogenic diets, respectively). Subjects ate 1.46 times greater mass of food on the lower-protein plant-based diet, indicative of compensatory feeding for protein, yet energy intake was lower than on the ketogenic diet. (*b*) Compilation of data from 44 published studies in which dietary per cent protein was varied relative to fat and carbohydrate combined, and subjects allowed to eat ad libitum. As per cent protein falls across the range seen in natural human diets (10–30%), food intake rises in compensation. As a result, protein intake is largely conserved (red points) but energy intake rises (blue points). The grey dashed lines indicate perfect protein leverage. Actual leverage (dashed blue line) was strong but incomplete (from [30] and [31]). (*c*) Means from the three randomized control trials to have tested protein leverage, compiled. As predicted by protein leverage, energy intake increased with declining per cent protein down to 10% protein, but this trend did not continue at 5% protein, indicative of a break point at protein concentrations below physiological requirements (figure 1).

Constancy is a hallmark of physiological regulation [11]. If intake of protein is both regulated and its regulation is prioritized over (i.e. stronger than) regulation of other dietary components, then excess energy intake will result when protein becomes diluted in the food supply by fats and carbohydrates [6] (figure 1*a*,*b*). Because protein typically comprises around 15% of total energy intake (e.g. [26,28]), regulating absolute protein intake requires that even a small decline in the proportion of protein in the diet ‘leverages' a disproportionately large increase in food intake (figure 2*a*). If protein is diluted with low-energy fibre and/or water, then such leveraging of food intake will not result in excess energy consumption, but when protein is diluted by energy-dense fats and carbohydrates, increased food intake will translate into excess calorie intake, with attendant increased risks of overweight and obesity (figure 2*b*,*c*).

There are thus three features necessary to demonstrate that protein leverage has contributed to variation in energy intake: (i) that the intake of protein is regulated, i.e. that there is a specific appetite for protein; (ii) that the regulation of protein is prioritized over the regulation of fat and carbohydrate intake (figure 1*a*,*b*); and (iii) that in relevant ecological settings variation in the dietary concentration of protein relative to fat and carbohydrate correlates negatively with energy intake. We discuss these in turn.

### (a) Specific appetite for protein

The concerted study of nutrient-specific appetites began with Curt Richter's work in the early 1900s [35], but the major focus in the field of appetite control has been on the control of energy intake rather than specific nutrients. Nonetheless, there is now abundant behavioural evidence that nutrient-specific appetites for protein, carbohydrate, fat and at least two mineral micronutrients, sodium and calcium, are widespread, being demonstrated in organisms spanning acellular slime moulds, insects, fishes and mammals (e.g. [14,36–40]). Laboratory experiments and field observations using nutritional geometry have shown that animals can regulate and defend multi-nutrient intake targets by a combination of selecting among foods differing in nutrient balance and adjusting the amounts of each food consumed [14,41].

Such studies indicate that nutrient-specific appetites work together to guide nutrient balancing in appropriate food environments, but also that nutrient-specific appetites and their mechanisms compete for access to the behavioural final common path [42] when the food environment constrains or subverts nutrient balancing. The perspective of competing nutrient-specific appetites casts a different light on phenomena such as leptin and insulin resistance in the brain. Where energy intake is excessive on low-protein, high-energy diets despite elevated anorexic signals such as leptin and insulin, perhaps this reflects neuronal competition from stronger protein signals, rather than a pathological failure of response to the ineffective signals; hence, ‘ignoring’ rather than ‘resistance’.

There has only been one experimental study designed using NGF principles to test for nutrient balancing in humans ([34], discussed further below), showing target regulation for protein and carbohydrate during a 3-day period in which subjects could select between an array of foods comprising three protein-to-carbohydrate ratios. Griffioen-Roose *et al*. [43] demonstrated in a randomized crossover experiment that a 14-day pre-treatment on a low-protein diet (5%) subsequently elicited specific selection of high-protein, savoury-flavoured foods from a large array of foods offered ad libitum, leading to a compensatory increase in protein intake without a change in total energy intake. This increased preference for savoury food cues after low-protein feeding was accompanied by increased brain activity, measured by functional magnetic resonance imaging, in the reward-related inferior orbitofrontal cortex specifically in response to stimulation with savoury food cues [44].

Whereas nutrient-specific appetites are well characterized behaviourally and appear to be universal, less is understood about their mechanisms, although this situation is changing with active research underway in model systems such as *Drosophila* and rodents. Mechanisms underlying nutrient-specific appetites involve both learned and innate responses, the latter including sensory modulation, dedicated central neural pathways and peripheral feedbacks (e.g. [39,40,45–48]).

Circulating levels of amino acids are the principal nutrient signals of the protein need state, acting via the brain [48–50]. Endocrine signals of protein state are also expected to play a role in protein appetite control, probably deriving from lean tissues and being related to some combination of protein breakdown and disposal [14,50]. The idea that a powerful and hitherto underappreciated appetite signal derives from lean mass is explored further by Hopkins *et al*. [51].

Fibroblast growth factor 21 (FGF21) was the first discovered endocrine signal of low-protein state (protein hunger) [52]. Circulating FGF21 is elevated in mice in response to protein restriction independently of total energy intake [53]. FGF21 is secreted primarily by the liver and crosses the blood–brain barrier where it acts centrally to regulate feeding behaviour and metabolic physiology [54,55]. Under pharmacological administration of FGF21, mice and rats increase total food intake on a fixed diet, and exhibit a shift in macronutrient selection when offered a choice of foods, selectively increasing protein intake and demonstrating reduced sweet preference (at least when animals are simultaneously carbohydrate replete) [56–63]). When mice can select between foods of different macronutrient compositions, FGF21 administration has little or no effect on total energy intake, indicating that increased intake on no-choice, low-protein diets is driven by compensatory feeding for protein, rather than being a secondary response to FGF21-induced elevated energy expenditure [57,58,62,64]. Rather, it would appear that elevation of energy expenditure is a mechanism for voiding excess ingested calories driven by protein leverage, thereby maintaining body composition despite hyperphagia.

Results from rodents are consistent with experimental and genome-wide association data in humans, showing that FGF21 is elevated under low-protein intakes (e.g. [52,54,65]) and that genetic variants are linked to macronutrient intake [66,67].

Whereas FGF21 is a likely candidate for a low-protein endocrine signal, its dose–response characteristics indicate that it cannot act as a signal for excess protein intake (protein satiety). This follows because FGF21 increases below a low-protein intake threshold, above which it is essentially absent in circulation (fig. 1C in [53]). Given that protein intake is regulated under both high and low-protein dietary conditions, it follows that there are other protein appetite signals responsible for limiting excess protein intake. Proteomic analysis guided by nutritional geometry in mice offers a means to identify potential candidates with suitable dose–response characteristics and macronutrient specificity [68]. Signals inhibiting high-protein intake may relate to the search for unknown signals inhibiting overfeeding in general, as discussed in this issue by Clemmensen and co-workers [69]. Studies have shown differential effects of ingested protein and carbohydrate on gastrointestinal hormones. Protein ingestion preferentially favours gastrin and cholecystokinin, whereas carbohydrate ingestion preferentially favours gastric inhibitory polypeptide and glucagon-like peptide 1 (GLP-1). Interestingly, GLP-1 (and GLP-1 agonists) in turn act on feeding centres in the brain, promoting hepatic FGF21 production via mechanisms including adrenal cortex production of glucocorticoids [70]. One potential candidate as a systemic protein satiety hormone is glucagon, which has been implicated in the control of macronutrient selection [71] and contributes to a liver-islet alpha cell axis for the control of plasma amino acid levels, in which a high plasma amino acid level induces alpha cell hyperplasia and, in turn, increased glucagon production to stimulate amino acid breakdown [72]. Another potential candidate is insulin, which like amino acids, acts on orexigenic neuropeptide Y/agouti-related peptide neurons to inhibit their activity [73].

### (b) Protein prioritization

There have been three randomized control trials (RCTs) explicitly testing for protein prioritization as a mechanism in leveraging energy intake in humans [32–34] (figure 2*b*,*c*). Additionally, an earlier 6-day, in-house, single-blind pilot study [74] provided results from 10 subjects that were consistent with protein leverage but did not attempt to disguise the nutritional composition of foods or match treatment menus for variety and palatability.

Gosby *et al*. [32] measured food intake and hunger ratings in 22 lean subjects studied over three, 4-day periods of single-blind in-house dietary manipulation at a Sydney University sleep centre. Each 4-day period provided a fixed menu containing 10%, 15% or 25% energy as protein (fat was fixed at 30% and carbohydrate was varied to maintain equivalent energy density), with these three macronutrient ratios allocated in random order to each participant. Menus comprised 28 foods, each of which had been designed in three versions (10%, 15% or 25% protein) that were matched for palatability [75]. Some foods were designed to be sweet and others savoury independently of macronutrient content. As a result, energy density, palatability, availability, variety and sensory quality were matched across macronutrient treatments. Lowering the per cent protein of the diet from 15% to 10% resulted in 12% higher total energy intake, a difference that was apparent from the first day of treatment and remained consistent thereafter (figure 2*c*). Tellingly, 70% of this increased calorie intake came from subjects snacking between meals, favouring savoury-flavoured over sweet-flavoured snack foods. Such protein-seeking behaviour on 10% protein was consistent with the results of Griffioen-Roose *et al*. [43,44] discussed above and was accompanied by increased circulating FGF21 [65]. This result illustrates the susceptibility to ‘protein decoys’—low-protein foods designed with savoury flavour characteristics which can trick people into ingesting calories while leaving the protein appetite unsatisfied [6].

Increasing protein from 15% to 25% did not result in a decline in energy intake (figure 2*c*), although on the fourth day of the trial there was a greater increase in the hunger score between 1 and 2 h after the 10% protein breakfast compared with the 25% protein breakfast. Even though a flattening of the relationship between intake and per cent protein is expected at higher values of per cent protein (owing to the nature of a power function; figure 1*b*), the lack of a detectable difference in intake between 15% and 25% was not as predicted.

Campbell *et al*. [34] elaborated upon the Sydney study design in a single-blind, in-house trial conducted at the University of the West Indies in Jamaica. Sixty-three adult survivors of marasmus and kwashiorkor were recruited to the 2-phase trial. In the first phase, participants were free to select for 3 days from foods containing 10%, 15% and 25% protein. Thirty-one culturally relevant experimental foods were designed, each in one of three macronutrient versions [74]. Subjects were then randomized in the second phase to one of three diets with protein fixed at 10%, 15% or 25% for 5 days. During the self-selection phase, both marasmus and kwashiorkor groups selected a similar diet composition, comprising 14.7% protein, which differed highly statistically significantly from the null expectation (16.7%) if subjects had chosen indiscriminately. This result indicates the selection of a target intake for both protein and carbohydrate (fat was fixed at 30% energy). In the second phase, energy intake increased with decreasing dietary per cent protein, being greatest for 10% protein, intermediate for 15% protein and, unlike in the Sydney trial, least for 25% protein diet (figure 2*c*). Again, survivors of kwashiorkor and marasmus could not be distinguished. Body weight changed across phase 2 as a positive function of energy intake.

Martens *et al*. [33] conducted a randomized, single-blind crossover study with 79 subjects provided with ready-made main meals eaten at the University of Maastricht and ad libitum access to low-protein (5%) snacks at home. Meals during each 12-day treatment period comprised 5%, 15% or 30% protein (fat was fixed at 35% of total energy) from dairy or plant sources. Irrespective of protein type, total energy intake was significantly lower in the 30% protein treatment, but the 5% and 15% treatments did not differ (figure 2*c*). The failure of subjects to increase energy intake on the 5% protein diet, despite presumably being protein-hungry [43,44], suggests that protein leverage does not operate at very low dietary protein concentrations. As discussed above, in the case of perfect leverage, maintaining absolute protein intake constant would require food intake to increase exponentially with decreasing per cent protein [6,7,24] which must inevitably impose a break point for the protein leverage response at some lower per cent protein. We have proposed that this threshold lies somewhere below 10% protein in humans, which coincides with the lowest level of protein seen in the diets of food-sufficient populations and below which protein intake is considered to be inadequate [76]. Similar break points at around 5% protein have been reported repeatedly for rodents (e.g. [77–80]).

Collectively, the results of the RCTs suggest that protein leverage occurs across the range from 10% to 30% protein, with 5% protein falling below the break point (figure 2*c*). Leverage for protein is not complete, with lambda values for a fitted power function being around −0.3 (a value of −1 indicating complete leverage) suggesting, as expected, that other factors interact with protein leverage to influence energy intake.

A common feature of the three studies is that intake responses to dietary protein were apparent within 24 h and remained consistent for at least 12 days (the longest period tested). In this respect, the results accord with an earlier study by Weigle *et al*. [81], in which 19 subjects were tested in sequence on a 15% protein weight maintenance diet for two weeks, a 30% protein diet for two weeks matched in calorie intake to the weight maintenance diet and then an ad libitum 30% protein diet for 12 weeks. Carbohydrate was fixed at 50% in all diets, which were also matched for energy density. Energy intake decreased within 24 h on the high-protein, ad libitum diet and remained depressed across 12 weeks, even though orexigenic signals were elevated (reduced leptin levels, increased ghrelin).

Although not testing protein leverage explicitly, numerous studies in addition to Weigle *et al*. [81] have manipulated dietary protein and estimated ad libitum intake using various experimental methodologies over periods from several days up to 12 months. In an apparent exception where energy intake did not vary inversely with dietary protein density Blatt *et al*. [82] measured intakes over 24 h of subjects fed two meals (lunch and dinner) manipulated to contain 10%, 15%, 20%, 25% or 30% energy from protein. The authors suggest that more sustained changes in protein may be needed to see the effects of adjusting protein intake, a conclusion consistent with the effect reported in other experimental studies.

To explore the relationship between dietary per cent protein and ad libitum intake more broadly, Gosby *et al*. [30] compiled data from 38 published experimental trials, comprising 116 dietary compositions. Collectively, these trials encompass variation in per cent protein spanning 8–54% of total energy, 2–72% carbohydrate and 11–66% fat. The compiled data provided an opportunity to describe the individual and interactive effects of dietary protein, carbohydrate and fat on total energy intake. After controlling for confounders such as experimental methodology, body mass index and sex of subjects, per cent dietary protein was negatively associated with total energy intake, irrespective of whether carbohydrate or fat was the diluent of protein. This relationship was strongly apparent from 10% to 30% protein, the range which includes all known healthy human diets. Further studies with 22 extra diet compositions were included in an updated analysis by Raubenheimer *et al*. [31] (figure 2*b*).

### (c) Variation in dietary protein

The third feature necessary for examining a role for protein leverage in energy intake—that ecological variation in the percentage of energy contributed by protein is negatively associated with energy intake—differs in an important respect from the previous two. Both the existence of a protein-specific appetite and its dominance (protein prioritization) are properties of the organism and best examined in controlled experimental settings using specifically manipulated dietary compositions. The question of how these properties engage with variation in real food environments cannot, however, be examined in laboratory settings alone, but requires studies of how appetite systems interact with the many dimensions of dietary and other sources of variation in relevant ecological settings.

Several studies have tested, or provided data capable of retrospectively testing, predictions of the protein leverage hypothesis (PLH) in ecological settings. The DioGenes and PREVIEW large randomized controlled trials demonstrated improved weight loss maintenance over a 24-week period and reduced hunger scores over 148 weeks, respectively, in individuals prescribed high-protein, low-glycaemic index diets [83,84]. Austin *et al*. [85] examined longitudinal trends in macronutrient and energy intakes using 24 h recall data from the USA National Health and Nutrition Examination Survey conducted in the periods 1971–1975 and 2005–2006. While not specifically testing PLH, the finding that dietary percentage energy from protein decreased and energy intake and adiposity increased over the survey period is consistent with the hypothesis [30]. Martinez-Cordero *et al*. [86] examined data for 2031 women from the Cebu Longitudinal Health and Nutrition Survey to test the prediction that protein intake remained more constant over time than fat and carbohydrate across five consecutive 24 h recall measures (1986, 1994, 1998, 2002 and 2005). As predicted, the rate of change in protein intake was lower than fat and carbohydrate, even when controlling statistically for household income and urbanicity index. Bekelman *et al*. [87] used cross-sectional data from 135 women in Costa Rica to examine whether protein leverage could explain socioeconomic (SES) variation in obesity. Consistent with PLH, absolute protein intake did not vary by SES; in middle and high-SES groups protein intake was less variable than fat and carbohydrate; and percentage of energy from protein (%EP) was inversely associated with total energy intake (TEI) across the full sample and among middle and high-SES groups. Not consistent with PLH, is there was no relationship between %EP and TEI among low SES women. However, variation in energy intakes was highest among low-SES women, as was the proportion who failed to meet protein requirements, leading the authors to suggest that food insufficiency might explain the difference in this group.

Recent studies have explicitly tested the prediction of PLH that energy intake varies inversely with dietary protein in population settings. Saner *et al*. [88] examined data for 137 adolescents with obesity from the Childhood Overweight BioRepository of Australia cohort. Results showed that total energy intake correlated negatively with dietary percentage protein, following a power function as predicted by PLH [6,7,24]. A similar analysis was done by Saner *et al*. [89], for a sample of children participating in the Physical Activity and Nutrition in Children (PANIC) study. In this case, data were collected longitudinally at average (s.d.) age 7.56 (0.4), 9.8 (0.4) and 15.8 (0.4). Dietary data taken from 4-day food records showed that at all time points energy intakes were inversely related to per cent energy from protein following power functions. Likewise, Blumfield *et al*. [90] showed in a prospective cohort study that protein leverage during pregnancy might explain differences in neonate body composition. Recently, Kebbe *et al*. [91] reported finding no strong evidence for protein leverage during pregnancy in another cohort study. However, this conclusion was not based on analysis of the relationship between energy intake and the proportion of energy as protein in the diet, as is required to test for protein leverage (see figure 1).

**Figure 3.**
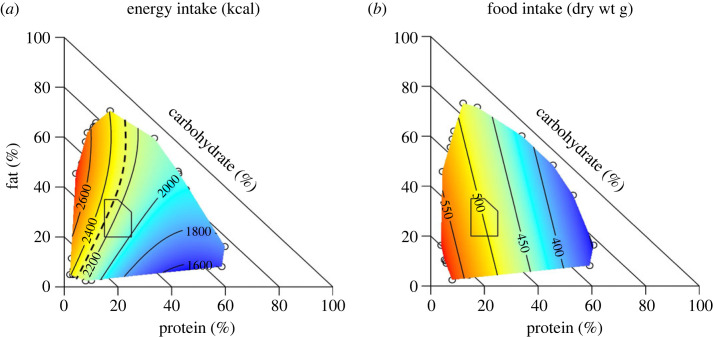
Right-angled mixture triangle showing relationships between variation in the three-dimensional macronutrient composition of daily diets of 9341 free-living adult Australians and energy intake (*a*) and food intake (*b*). In these plots, dietary per cent carbohydrate increases from the diagonal that connects 100% fat with 100% protein (which represents 0% carbohydrate) to the origin (100% carbohydrate) [92]. The dashed line in (*a*) represents the estimated average energy requirement of respondents, delineating diets predicted to be associated with positive (left) and negative (right) energy balance. The superimposed polygon shows the acceptable macronutrient distribution range recommended in the diets of Australians (from [13]).

Grech *et al*. [13] performed a more detailed ecological analysis on the Australian National Nutrition and Physical Activity Survey using proportions-based nutritional geometry, which allows the effects of variation in three-component macronutrient mixtures to be examined in two-dimensional plots [92,93]. Energy intake increased with decreasing dietary protein, as predicted by PLH (figure 3*a*). This effect was strongest on diets in which fat diluted protein, suggesting that high energy density might interact with protein leverage to drive excess energy intakes. Energy density alone cannot account for high energy intakes, however, because energy intakes increased with decreasing protein also on diets low in fat where the predominant macronutrient diluent of protein was carbohydrate (fig. 2C in Grech *et al*. [13]). Furthermore, the dry weight of food eaten also increased with decreasing dietary protein (figure 3*b*), which as explained above is indicative of protein leverage. These results suggest that protein dilution leverages food intake, and the consequences for energy intake are dependent on both the percentage protein and energy density of the diet.

The same effect was seen in a recent human clinical trial comparing a low-fat, low-energy density (0.96 kcal g^−1^), plant-based diet with a high-fat, high-energy (1.9 kcal g^−1^) animal-based ketogenic diet [29] (figure 2*a*). In terms of per cent energy, protein was matched at approximately 14.5% between the diets, but because of the difference in energy density between the two diets, protein concentration differed per gram of food (3.5% protein by wet weight in the plant-based diet versus 7.5% in the ketogenic diet). Indicative of protein leverage in response to this dilution of protein within the food matrix, ad libitum food intake was 1.45 times (667 g) higher on the plant-based diet, yet owing to the lower energy density, total calorie intake was lower on the low-fat diet. Had food intake doubled on the plant-based diet (i.e. 1.3 kg more food eaten each day), protein and energy intakes would have been the same as on the ketogenic diet. Because food intake did not double, energy intake was greater on the ketogenic than the plant-based diet, providing an important illustration of how protein leverage and dietary energy density interact. A similar phenomenon has been reported in rodents [94], where, for example, dilution of protein with indigestible fibre stimulates a compensatory increase in food intake without a commensurate increase in energy intake, whereas dilution of protein by fat results in increased food intake and maximal energy intake.

Since the analysis of diet surveillance and other sources of population dietary data continues to attract criticism, sometimes vehemently, it is worth mentioning the limitations and benefits of ecological studies such as those discussed above. A common error is to infer that such studies are weak attempts at testing for protein leverage because observational data can only establish correlation, not causation, and population data are riven with confounds. This is true, but we stress that the primary purpose of these studies is not to *test* protein leverage; that is done in experimental settings (as above). Their purpose is to test predictions of the specific hypothesis that *in relevant ecological settings* protein leverage contributes to variation in energy intake (the PLH), and identify potential mediators, moderators and modulators (e.g. energy density, other dietary components, physical activity, food insecurity [95]) for further examination in controlled experimental settings. It is, nonetheless, true that detecting the predicted relationship between dietary protein density and energy intake does supplement evidence from RCTs for the existence of protein leverage, especially if potential confounds are critically examined using statistical and modelling procedures (e.g. [96]).

Perhaps the greatest benefit of population studies is that they enable potential ecological drivers and interventions to be identified, for example, the foods responsible for diluting dietary protein and the environmental factors that lead to excessive consumption of such foods.

## 4. Ecological analysis: what is causing protein dilution?

**Figure 4.**
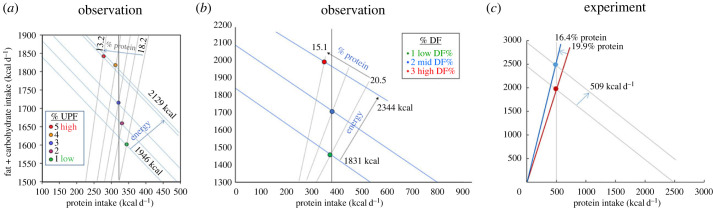
Relationships between intake of highly processed foods, dietary macronutrient composition and energy intakes from diet surveillance studies (*a*,*b*) and a randomized control trial (*c*). (*a*) Respondents in the 2009–2010 NHANES cycles were partitioned into quintiles based on per cent contribution of ultra-processed foods (UPF) to daily energy intake. With increasing UPF, percentage protein decreased, fat, carbohydrate and energy intake increased, but protein intake varied little, as predicted by protein leverage (replotted from tables 2 and 3 in [97]). (*b*) Equivalent analysis of the Australian Adult Health and Nutrition Survey, in which respondents were partitioned into tertiles of discretionary food intake (modified from [13]). (*c*) Geometric plot of data from an inpatient randomized control trial in which subjects had ad libitum access to ultra-processed or unprocessed diets for 14 days. Diets were of equal caloric density (data are replotted from fig. 2 in [98]). Overall, subjects ingested a lower per cent protein and higher energy intake on the ultra-processed diet, yet absolute protein intake was the same.

Two recent studies have tested the predictions of PLH in population settings and simultaneously examined potential ecological drivers. Martínez-Steele *et al*. [97] analysed NHANES 2009–2010 dietary recall data to test the hypothesis that consumption of UPF is associated with dietary protein dilution and drives energy over-consumption via protein leverage. Results showed that dietary protein decreased across quintiles of increasing per cent UPF contribution to daily energy intake, supporting the prediction that highly processed industrial foods are associated with the dilution of dietary protein with fats and carbohydrates (figure 4*a*). Absolute macronutrient intakes tightly conformed to the predictions of PLH, with protein varying little but fat, carbohydrate and total energy increasing with decreasing percentage energy from protein.

**Figure 5.**
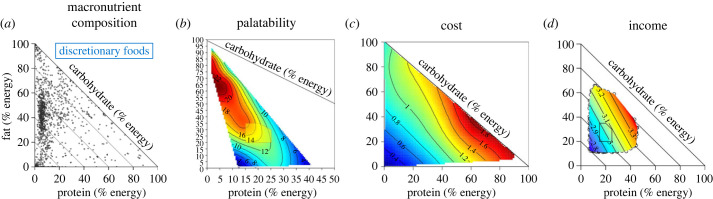
Potential biological (*a*,*b*) and socioeconomic (*c*,*d*) influences on processed food consumption. (*a*) Discretionary foods cluster in a low protein region of three-dimensional macronutrient space, hence are susceptible to over-consumption via protein leverage (from [13]). (*b*) Response surface generated from hedonic ratings of milk-based mixtures varying in fat, carbohydrate and protein. Results show a close correspondence between the composition of discretionary foods (*a*) and peak palatability. Recalculated and plotted in three macronutrient dimensions from an experiment designed to assess the effects of variation in fat and sugar ratios [99]. (*c*) Protein is expensive—the dollar cost of foods increases with increasing protein content and is lowest for diets low in protein and high in carbohydrates (blue region) (replotted from [100]). (*d*) The correlation between family incomes and dietary macronutrient compositions shows lower incomes are associated with diets lower in protein (as expected from (*c*)) and higher carbohydrate proportions, possibly explaining associations between socioeconomic status and obesity incidence. Data from the Australian National Nutrition and Physical Activity Survey.

Similar results were obtained in the analysis mentioned above of 24 h recall data from the Australian National Nutrition and Physical Activity Survey [13] (figure 4*b*). This analysis, however, went further, using mixture geometry to construct a model that examined relationships between the macronutrient compositions of specific food groups, dietary macronutrient ratios and energy intakes. Strikingly, the macronutrient compositions of discretionary foods (the equivalent of UPF in the Australian diet classification system) (shown in figure 5*a*) conformed closely with the compositions of the diets associated with maximum energy intake (figure 3*a*)—having low-protein and intermediate fat-to-carbohydrate ratios. Examination of the separate contributions to energy intake of discretionary foods and foods that fall within the ‘five food groups' recommended in the dietary guidelines showed that the peak in total energy intake was specifically associated with high intakes of discretionary foods, with the relative contribution to energy intake of the ‘five food groups' being low in that region of the macronutrient space.

Overall, these results implicate highly processed foods as drivers of increased energy intake via their effect on protein dilution and protein leverage, in interaction with factors such as palatability and energy density. While there has been no study that explicitly tested this association in an experimental setting, one randomized control trial compared energy intakes and weight gain associated with a 14-day exposure to an ultra-processed diet or a whole food diet partly matched for macronutrients and presented calories [98]. Consistent with the population studies, results showed that calorie intakes and weight gain were higher on the processed than the whole food diet. Although the study was not designed to test the mechanisms involved, the fact that protein intakes did not differ, but fat and carbohydrate intakes were higher on the ultra-processed diet, led the authors to suggest that protein leverage could partially explain the results (up to 50% of the increased intake on the ultra-processed diet treatment). This conclusion was based on the composition of the experimental diets as presented to the subjects (14 versus 15.6%). However, participants actually consumed 16.4 versus 19.9% protein of total energy and virtually the same absolute intake of protein (figure 4*c*, from fig. 2 in [29]). As the authors indicate, the unanswered question is why did subjects ingest a different per cent protein to that provided in the menus? This must indicate the selection of foods from within meals and perhaps the contribution of snack foods offered. If subjects were relatively more prone to selecting palatable, lower-protein items within the ultra-processed diet, or higher-protein items within the whole food menus, this would exacerbate the per cent protein difference between the treatments and engage protein leverage more strongly. Our point is that protein appetite, protein leverage and other factors such a palatability interact, potentially yielding positive feedbacks driving excess energy intake. A difference in energy density between the treatments was not a factor in this experiment, but it too would interact and amplify the effects of protein leverage, as discussed above.

Having identified the food groups implicated in driving energy over-consumption, the next step is to examine why these foods are eaten over alternatives that support balanced, healthier, diets. Several reasons have been suggested, including their aggressive marketing, convenience, relatively cheap price and the fact that they are engineered to be ‘hyperpalatable’. Such factors can readily be integrated into geometric models, as illustrated in figure 5.

## 5. Moving targets: using protein leverage to generate new hypotheses

Among the benefits of integrative models is that they provide a framework for going beyond existing data to generate new testable hypotheses for unexplained phenomena, even resolving apparent paradoxes. To this point, we have discussed the dilution of protein in the diet as a driver which interacts with dietary energy density to explain excess energy intake. We next show that by incorporating differences in protein and energy requirements and levels of energy expenditure, protein leverage theory can potentially illuminate several unexplained phenomena.

**Figure 6.**
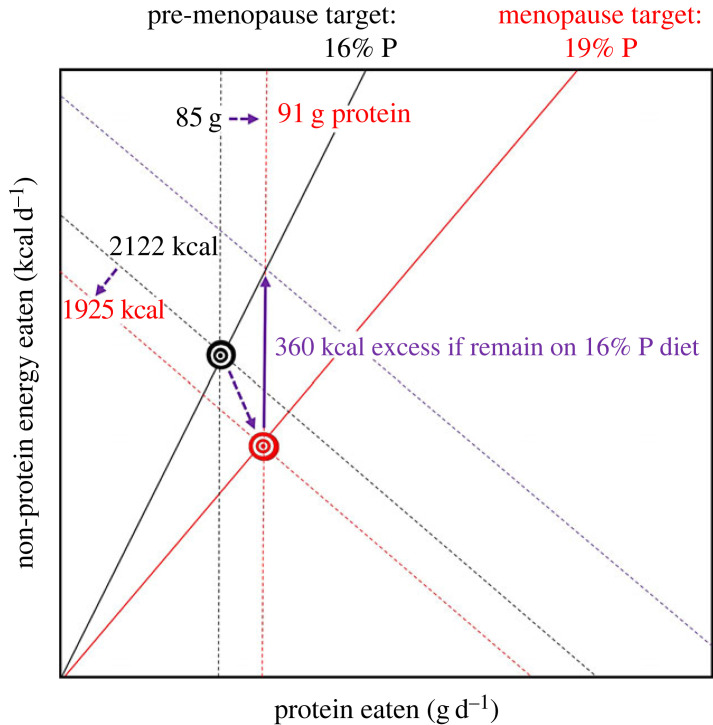
Graphical model illustrating the implications of the shift in protein and energy requirements during the menopause transition, parameterized using data from the literature. The black bullseye illustrates daily target intakes for protein and non-protein energy prior to menopause. The red bullseye indicates an increased need for protein during the menopausal transition (7%) accompanied by reduced energy requirements (9%) owing to a reduction in physical activity. Simultaneously meeting the higher-protein target while remaining in energy balance requires shifting to a higher per cent protein diet (red radial), whereas reaching the protein intake target while remaining on a 16% protein diet requires ingesting excess calories (purple arrow) (from [101]).

Because of the power law relationship between dietary per cent protein and food intake (figure 1*b*), protein leverage is amplified if the protein target increases relative to non-protein energy (nPE) requirements. This is because, as the protein target increases linearly, the number of extra calories that must be eaten to attain the higher target on a given low-protein diet will increase exponentially. For example, a disproportionate increase in protein to nPE requirements may explain body weight gain, increased fat mass and decreased lean body mass with age in both sexes [15] and across the menopause transition in women [101] (figure 6). Ageing and menopause are associated with reduced skeletal muscle mass and net bone resorption, increased protein catabolism, impaired protein synthesis and elevated FGF21.

Additionally, if obesity results in increased protein requirements and/or reduced energy expenditure, then protein leverage theory predicts that susceptibility to obesity will accelerate unless dietary macronutrient ratios change commensurately towards a higher protein: nPE. This might provide a mechanism to explain the observation that as body fat increases it becomes increasingly difficult to lose weight and sustain weight loss (e.g. [102]).

There is, indeed, evidence that protein requirements increase disproportionately relative to energy requirements with obesity and the metabolic syndrome and are associated with elevated FGF21 [103]. Insulin resistance disinhibits protein catabolism in muscle and hepatic gluconeogenesis, both of which result in decreased protein efficiency and thus a need to eat more protein to meet requirements. The kidneys provide another route for protein loss via proteinuria and albuminuria, which are associated with obesity and metabolic syndrome [104,105]. Elevated FGF21 accompanies chronic kidney disease, increasing during its early stages and predicting progression in diabetes [106].

Another circumstance in which there may be a relative increase in protein to nPE requirements is when there is a decline in energy expenditure accompanying a change in lifestyle, as seen particularly markedly in retired athletes (e.g. [107]) and more generally in young adults transitioning to less active lifestyles. The protein leverage effect would be exacerbated if in addition to reduced energy expenditure protein needs are set higher in response to a habitually high-protein diet or an anabolic exercise regime.

An increased protein requirement might reflect diet during early life and even preconception. High-protein infant formula feeding increases susceptibility to obesity in subsequent years [108–110], an effect we hypothesized might reflect protein leverage interacting with the early development of an increased protein requirement in a low-protein, high-energy food environment [111]. As discussed above, based on analyses of population cohorts, children and adolescents do appear to show protein leverage [88,89].

Yet another source of variation in protein requirements arises from the gut microbiota. Some bacterial species serve as a net source of essential amino acids in the host system, whereas other species use protein as an energy source and thus act as a protein sink, thereby increasing host protein need and resulting in elevated FGF21 [112,113]. Elevated intake of dietary fibre has the potential to increase the net activity of amino acid-producing bacterial assemblages [113], providing another potential mechanism linking protein leverage and a diet high in fibre-poor, industrially processed foods (see above).

Protein leverage may drive excess energy intake on a low-protein, high-energy diet, but not everyone gains weight under excess energy intakes, nor retains excess weight when returned to energy balance after experimental overfeeding [114]. Indeed, voiding excess ingested energy is part of the homeostatic control of weight [115] and was apparent at a population level in a cohort of predominantly healthy, lean children demonstrating protein leverage [89]. Genetic factors play a role in increasing the susceptibility to weight gain with overfeeding, as does insulin resistance and previous obesity [1,114], but the mechanisms are not fully understood. Variation in genetic loci involved in protein metabolism and signalling, including FGF21, would be expected to associate with variation in obesity risk, both between individuals within a population and between populations as a function of ancestral diet [6,111].

## 6. Conclusion

Obesity is a complex multifactorial condition that arises from the dynamic interaction of biology and psychology with food environments. We believe a priority is to identify the most important sets of interactions for advancing the understanding of obesity and moving closer towards solutions. We have reviewed a body of work that has applied nutritional geometry, a tool for examining nutrient–nutrient interactions and their interface with biology and food environments, to examine the problem. This work suggests an integrative model of obesity, at the centre of which is the acute sensitivity to protein deficiency or amino acid imbalance that humans share with many species. Protein imbalance is transduced into corrective responses via signalling molecules such as FGF21 which, behaviourally, triggers protein seeking through increased protein appetite. If high-protein foods are eaten, the protein imbalance is redressed and FGF21 release is reduced. By contrast, if low-protein foods are engaged, there is a compensatory increase in food intake to reduce the protein deficit, which results in increased incidental intake of the components diluting protein. Where the diluents are non-caloric, such as water or fibre, there is no adverse impact on energy balance. By contrast, where protein is diluted by fats and carbohydrates, energy intake increases, but is offset by a compensatory increase in FGF21-triggered energy expenditure. However, where low-protein diets are simultaneously high in fats and carbohydrates and low in non-caloric diluents such as fibre and water, the homeostatic mechanisms can be overwhelmed leading to positive energy balance, particularly where carbohydrates are rapidly digested non-resistant starches and sugars. If exposure is chronic, this leads to increased adiposity which itself can exacerbate the imbalance by decreasing protein efficiency thus increasing its deficiency and triggering further intake—a positive feedback. Other causes of decreased protein efficiency, such as occurs in the menopausal transition and possibly through early exposure to high-protein diets such as some processed infant formulae, can likewise cause or contribute to positive energy balance.

This biological model aligns closely with epidemiological associations of positive energy imbalance and highly processed industrial foods, which typically are high in fats and simple carbohydrates relative to protein, with high energy density. Simultaneously, these products have properties that influence the likelihood that they will be chosen over healthier alternatives, such as umami flavouring (savoury snacks), hyper-palatability and low cost. In integrating across levels from molecular signalling to global food systems, this model exposes a network of interactions which collectively explain and predict the incidence and circumstances of increased risk of obesity. Ultimately, this systems perspective suggests that the excessively high incidence of obesity is as much an issue of imbalances in societal systems as imbalance in biological systems. The challenge ahead is to identify intervention points in the complex system of factors that tip the balance towards excessive consumption of obesogenic diets, such as ubiquitous exposure highly processed foods.

How broad do we go? For problems like obesity, which pay no respect to conventional disciplinary boundaries, we need to do likewise and broaden the scope from human health to the health of the planet on which our health ultimately depends. Recent studies, not discussed in this review, have begun this, by integrating nutritional geometry with input–output analysis to examine the simultaneous effects of various diets on nutritional, environmental and economic indicators [116]. There, too, the protein appetite has proven to be central, for example in directing diets towards increasing consumption of animal proteins which are associated with high global greenhouse emissions. Less obviously, the models have demonstrated that reducing animal proteins is likely to reduce emissions only in some circumstances, depending on what they are replaced by. If they are replaced by highly processed industrially manufactured foods, the emissions associated with the production costs of excess energy consumed due to protein leverage cancel out the gains from reducing animal protein. Ironically, studies suggest that increasing atmospheric carbon dioxide reduces both protein and fibre relative to carbohydrate in many crop plants—the same effect as ultra-processing—potentially providing another positive feedback that could exacerbate the problem [111].

In conclusion, it is only through situating specific nutrients and biological factors within their broader context that we can hope to identify sustainable intervention points for slowing and reversing the incidence of obesity and associated complications. An immense amount of knowledge exists around relevant factors but attempts to synthesize this information into evidence-based coherent models are in their infancy. For that, a diversity of perspectives is needed, such as shown at this meeting, and systems perspectives to examine the connections and interactions among these factors.

## Data Availability

This article has no additional data.
